# Supplementary material for: Integrated analysis of the aqueous humor microbiome and lens capsule transcriptome in high myopia cataract: a pilot study
Source: Front Med (Lausanne). 2026 Jun 16;13:1845205. doi: 10.3389/fmed.2026.1845205 (PMC13314463; doi:10.3389/fmed.2026.1845205)
Supplement: Supplementary file 3 [file Data_Sheet_1.zip › 7.Average/1.Community_Structure/heatmap/C372089/Species_top30_nocluster.pdf]

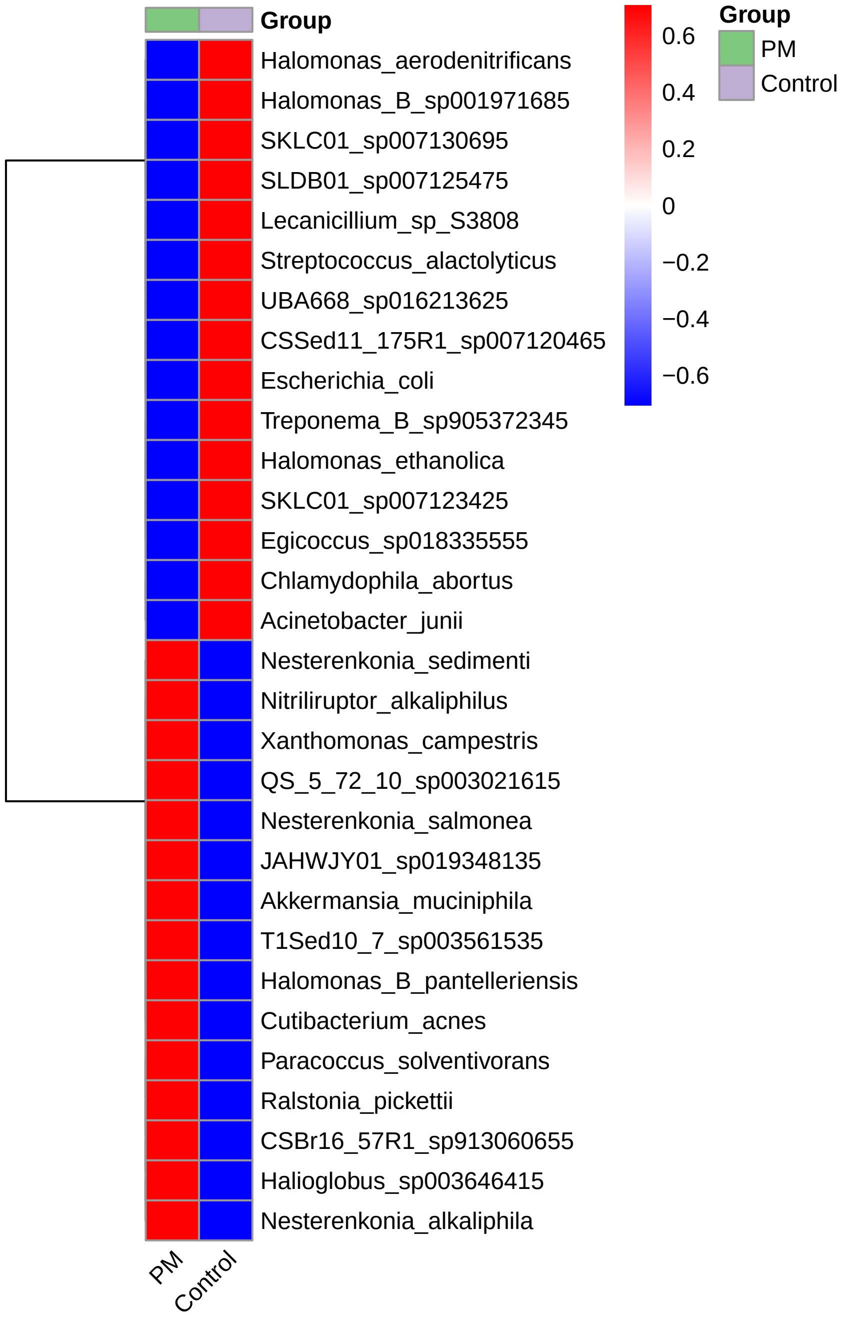
Group
Halomonas_aerodenitrificans
Halomonas_B_sp001971685
SKLC01_sp007130695
SLDB01_sp007125475
Lecanicillium_sp_S3808
Streptococcus_alactolyticus
UBA668_sp016213625
CSSed11_175R1_sp007120465
Escherichia_coli
Treponema_B_sp905372345
Halomonas_ethanolica
SKLC01_sp007123425
Egicoccus_sp018335555
Chlamydophila_abortus
Acinetobacter_junii
Nesterenkonia_sedimenti
Nitriliruptor_alkaliphilus
Xanthomonas_campestris
QS_5_72_10_sp003021615
Nesterenkonia_salmonea
JAHWJY01_sp019348135
Akkermansia_muciniphila
T1Sed10_7_sp003561535
Halomonas_B_pantelleriensis
Cutibacterium_acnes
Paracoccus_solventivorans
Ralstonia_pickettii
CSBr16_57R1_sp913060655
Halioglobus_sp003646415
Nesterenkonia_alkaliphila
PM
Control
0.6
0.4
0.2
0
−0.2
−0.4
−0.6
Group
PM
Control
